# On the Improvement of Default Forecast Through Textual Analysis

**DOI:** 10.3389/frai.2020.00016

**Published:** 2020-04-07

**Authors:** Paola Cerchiello, Roberta Scaramozzino

**Affiliations:** Department of Economics and Management, University of Pavia, Pavia, Italy

**Keywords:** text analysis, credit scoring, default, classification models, finance

## Abstract

Textual analysis is a widely used methodology in several research areas. In this paper we apply textual analysis to augment the conventional set of account defaults drivers with new text based variables. Through the employment of *ad hoc* dictionaries and distance measures we are able to classify each account transaction into qualitative macro-categories. The aim is to classify bank account users into different client profiles and verify whether they can act as effective predictors of default through supervised classification models.

## 1. Introduction

The change in all sectors of the economy that we are witnessing in recent years is so rapid that it speaks of the fourth industrial revolution. In the era of big data, across all sectors companies' main asset has become data. There is an increasing use of data, as the use of digital technologies increases, the amount of information collected increases exponentially. As a result, firms sit upon swathes of data, but the key is being able to derive value from them. In the financial sector, data are used for multiple purposes, one of which is credit scoring. This refers to the techniques used to assign creditworthiness to a customer to be able to distinguish “good” from “bad” customers i.e., clients who will repay their financing and those that will be insolvent. The probability that an applicant is insolvent is determined by analyzing the information on the latter (Hand and Henley, 1997). Credit scoring models are fundamental for banks to guarantee a correct forecast of default risk for financed loans, which translates into a reduction in losses and an increase in profits. There are numerous techniques for this purpose (Efron, 1979; Baesens et al., 2003; Jayasree and Balan, 2013; Emekter et al., 2015). Although nowadays most of the models in question use quantitative information, typically financial data, the latter is no longer sufficient to properly profile customers in a world that is now increasingly digital. This type of information, called hard information, is contrasted by another category of so-called soft information. Soft information is the term used to indicate information obtained through textual analysis, in this case, we talk about unstructured data. Text mining arises in this context. Text mining is the mechanism for extracting relevant information from unstructured documents to discover unknown patterns (Gupta and Lehal, 2009; Aggarwal and Zhai, 2012).

Even in today's standards, the traditional approach, which uses only hard information, is that which is widely used by firms but there is lack of studies that analyze textual information (Fei et al., 2015; Allahyari et al., 2017). Jiang et al. (2018) demonstrate how the use of textual data can increase the predictive power of a model, combining soft information with typically financial information analyzing the main p2p platforms in China. Groth and Muntermann (2011) state that exposure to intraday market risk management can be discovered through the use of text mining. Chan and Franklin (2011) show, through the use of textual data, that the forecast accuracy of their model improves similar traditional models by 7%. The advantages and disadvantages of Hard Information are analyzed by Liberti and Petersen (2018) who examine how information influences financial markets. Cornée (2019) demonstrates the importance of textual information for credit scoring by analyzing 389 presences of a French bank. Grunert et al. (2005) analyzing German SMEs, compare a model based on financial data, one on textual data and one mixed demonstrates how the latter is the best in terms of predicting loan defaults.

In this paper, we propose a credit scoring model that utilizes text mining. The variables extracted through textual analysis are used as predictors in the model. To extract this information we have classified the bank transactions into macro-categories and then considered the frequencies of each macro category and the total amounts. We then compared the classical model based on financial information and the one with the addition of variables derived from textual analysis. The rest of the paper is organized as follows: in section Methodology the methodology used is shown, in section Data we analyze the data, in section Results we report the results obtained and finally the paper is discussed and future research presented.

## 2. Methodology

In this section we explain the methodology used. We developed a default risk prediction model by combining financial information and textual information. The first step of the analysis was the extraction of relevant variables from the texts provided in the transactions. The method consists of 2 parts: pre-processing and knowledge extraction. The first one needed a lot of work and demanded most of the time spent on the analysis. Preprocessing plays a significant role in Text mining. Any task of text mining fully depends on the preprocessing step. High-quality of preprocessing always yielded superior results (Kumar and Ravi, 2016). In the preprocessing, we have cleaned up the texts of the transactions. We have created the textual corpus starting from the original text through the removal of stop words (typical and specific for the context), tokenization, removal of errors, and stemming. We then created the document term-matrix. The matrix describes the frequency of the terms in the documents. In our case, the rows represent the transactions and the columns correspond to the terms. The columns are extracted through the analysis of the corpus regarding the dictionaries, these were created manually in terms of key-value: by inserting the subject of the service as a key and the category of the transaction as value. The final goal of text mining was to obtain topics (macro-areas of interest relating to transactions) to create new variables for each one created and to insert these variables in the credit scoring model. The first problem encountered is due to spelling errors, due to this problem many transactions were not found in dictionaries. This obstacle is because the transaction descriptions are handwritten by the bank operators, who often abbreviate the words or write absently without paying attention. To overcome such issue we, therefore, decided to use a distance measure, the Levenshtein one, to attribute the word to the closest transaction under consideration (Levenshtein, 1966). This is a measure accounting for the difference between two strings, which is the minimum number of changes necessary to transform one word into another. Mathematically:

(1) $$\begin{array}{l} lev_{a,b}\left(i,j\right)\\ =\{\begin{array}{cc} max\left(i,j\right) & \text{if }min\left(i,j\right)=0,\\ min\left\{\begin{array}{c} lev_{a,b}\left(i-1,j\right)+1\\ lev_{a,b}\left(i,j-1\right)+1\\ lev_{a,b}\left(i-1,j-1\right)+1_{\left(a_{i}\neq b_{j}\right)} \end{array}\right\} & \text{otherwise.} \end{array} \end{array}$$

The algorithm flow, accordingly, consists in:

1. Pre-processing on the single transactions and creation of a textual corpus;
2. Search in the dictionary of the key corresponding to the clean string obtained from preprocessing;
3. Assignment of the value corresponding to the key of the dictionary, that identifies the category. If this is not found, the Levenshtein distance of the string is calculated from all the keys in the dictionary and the nearest dictionary value is assigned, with a maximum threshold of 10. This means that whether the distance is greater, neither category will be assigned (at the end of the investigation, the percentage of Na is around 15%);
4. After assigning a category to each transaction of the dataset, we created the variables to be included in the credit scoring model.

The categories have been grouped into macro-categories, the macro-categories chosen for the analysis are 5:

- Non-essential goods: including expenses for goods such as shopping, travel and living.
- Essential goods: including expenses in markets, pharmacies.
- Financial services and utilities: including expenses related to banks, payment services, telco companies, petrol stations.
- Revenue: including incomes such as transfers and dividends.
- Salaries: including wages and pensions.

For each of the previous 5 variables, we have therefore created 2 further variables: frequency and the total amount spent by the client for the specific category. We have thus obtained 10 new variables through the processing of textual data.

The second step of the analysis is the application of credit scoring model. The textual based categories created were added to the financial ones and the new dataset was used in the model. The chosen model is the lasso logistics. Lasso logistic model is a shrinkage method that allows obtaining a subset of variables that are strongly associated with the dependent variable, through regularization of the coefficients bringing them to values very close or even exactly equal to zero. Since the L1 penalty is used, the variables with a coefficient equal to zero are excluded from the model (Hastie et al., 2009). Mathematically:

(2) $$\begin{array}{l} L_{lasso}\left(\hat{\beta }\right)=\overset{n}{\underset{i=1}{\sum }}{\left(y_{i}-x_{i}^{\prime }\hat{\beta }\right)}^{2}+\lambda \overset{m}{\underset{j=1}{\sum }}|{\hat{\beta }}_{j}|. \end{array}$$

where *y*_*i*_ are the n-observations for the target variable (default/no default), *x*_*i*_ are the n-observations for the covariates, λ is the penalization parameter chosen by cross validation and β are the coefficient of the model.

Along with Lasso we fitted Elastic net as well. Since there were no statistical significant differences, we preferred to focus on Lasso because of an easier interpretation of the results. Before applying the lasso logistic algorithm, we pre-selected the relevant variables through the Kruskal–Wallis test. We decided to apply this test due to the size of the dataset: too few observations (400) with respect to the number of available variables (52). When the size of available data is limited, as in our case, Lasso can be not efficient enough in fitting parameters. Lasso is good at dropping out not significant variables if it can use an appropriate number of observations compared to the number of variables. Thus, we pre-selected the most relevant variables (without being too restrictive) through Kruskal–Wallis paying attention to the division in training and test. Kruskal–Wallis is a non-parametric method (no assumptions on the distribution of the data) that states if there is a significant difference between the groups. The null hypothesis states that the *k* samples come from the same population and the alternative hypothesis states that they come from different ones (Siegel and Castellan, 1988).

The KW test (Conover, 1971) is the non-parametric version of the well-known ANOVA test and represents a multivariate generalization of the Wilcoxon test for two independent samples, that can be adapted to our problem as follows. On the basis of C independent samples (each containing the transactions of a client) of size *n*_1_, *n*_*C*_ (the frequency of transactions for each client), a unique large sample *L* of size $N=\overset{C}{\underset{i=1}{\sum }}n_{i}$ is created by means of collapsing the original *C* samples. *L* can be organized as a matrix that contains a number of rows equal to *N* and a number of columns equal to *W* (the number of variables). Each entry of the matrix contains the frequency count of a specific variable along with each transaction. The *K*W test is then applied columns, in order to evaluate the discriminant power of each variable with respect to the client classification task. For each variable, the frequency vector corresponding to each column of the matrix *L* is ordered from the smallest frequency value to the largest one, and a rank is assigned to each transaction in the sample accordingly. Finally, one should calculate *R*_*i*_ as the mean of the ranks in each of the *C* original clients categories samples. The multivariate *KW* test can then be shown to take the following form:

(3) $$\begin{array}{l} H=\frac{12}{n\left(n+1\right)}\begin{array}{c} \sum_{i=1}^{C}n_{i}\frac{R_{i}^{2}}{n_{i}}-3\left(n+1\right) \end{array} \end{array}$$

After having selected only the significant variables, we applied the lasso logistics comparing the model keeping only the financial variables, the model with only the textual variables and the one obtained by the combination of the variables. For each analysis, the dataset was divided into 2 parts, 70% for training and the remaining 30% for the test. In addition, the model has been cross-validated using 5 folds. We applied the cross validation in the training set and then validated in the test set.

The comparison of the 3 models is based on mean misclassification error (mmce), area under the curve roc (auc), accuracy (acc), and roc curve (Krzanowski and Hand, 2009; Agresti and Kateri, 2011). Mmce is a prediction error metrics for a binary classification problem. The Roc curve is a graphical representation, along the two axes we find the sensitivity and 1-specificity, respectively represented by True Positive Rate and False Positive Rate. It is, therefore, the true positive rate as a function of the false positive rate. AUC is the area under the Roc curve, an aggregate measure of performance across all possible classification thresholds. Accuracy is the degree of correspondence of the estimated data with the real one.

## 3. Data

We have undertaken this analysis starting from 2 datasets: loans and transactions relating to an Italian bank. The paper was executed in collaboration with Moneymour. Moneymour is a FinTech startup that offers a payment method to provide instant loans for online purchases. It allows client to buy immediately and pay in installments.

In the former the original variables were:

- Date of the loan request,
- Loan ID,
- Default status,
- Amount,
- Number and amount of loan payments.

In the latter, there were:

- Client,
- Accounting date,
- Value date,
- Transaction amount,
- Reason code and reason text.

This study analyzed 164931 transactions and 400 loans from 2015 to 2018.

The financial variables extracted are:

- Sum of income,
- Sum of outcome,
- Average income,
- Average outcome,
- Number of income,
- Number of outcomes,
- Total number of movements,
- Sum of salary and average salary.

All listed variables referring to the first month, three months, six months and the previous year respectively to the request for financing, and financing obtained.

Summary statistics of financial variables are reported in Table 1.

**Table 1:** Descriptive financial features.

	**prev. funding**	**num. rev. month 1**	**num. rel. month 1**	**num. mov. month 1**	**num. rev. month 3**	**num. rel. month 3**	**num. mov. month 3**	**num. rev. month 6**	**num. rel. month 3**	**num. mov. month 6**	**num. rev. month 12**	**num. rel. month 12**	**num. mov. month 12**
Min.	0.00	0.00	0.00	0.00	0.00	0.00	0.00	0.00	0.00	0.00	0.00	0.00	0.00
1st Qu.	0.00	0.00	0.75	1.00	0.00	3.00	4.00	1.00	4.00	6.00	1.00	4.00	6.00
Median	0.00	1.00	6.00	7.00	3.00	16.50	21.00	6.00	34.00	39.00	11.00	55.00	71.00
Mean	0.23	1.46	13.78	15.23	4.22	41.38	45.59	8.10	81.39	89.50	14.93	150.50	165.40
3rd Qu.	0.00	2.00	23.25	26.00	6.00	74.25	83.25	12.00	149.25	165.20	22.00	248.20	271.80
Max.	0.00	9.00	90.00	93.00	43.00	253.00	265.00	59.00	466.00	485.00	97.00	873.00	911.00

Through the use of text mining, the transactions carried out by each client were analyzed and the new variables were created:

- Salary,
- Total output non-essential goods,
- Total output essential goods,
- Total financial services and utilities,
- Total salaries,
- Total income,
- Frequency output non-essential goods,
- Frequency output essential goods,
- Frequency financial services and utilities,
- frequency salaries,
- Frequency income.

Summary statistics of textual variables are reported in Table 2.

**Table 2:** Descriptive textual features.

	**Salary**	**Total output essential goods**	**Total financial services and utilities**	**Frequency output non essential goods**	**Frequency output essential goods**	**Frequency financial services and utilities**	**Frequency salaries**	**Frequency income**
Min.	0.00	-31084	-224137.0	0.00	0.00	0.00	0.00	0.00
1st Qu.	0.00	-1862	-19456.8	3.75	0.00	0.00	0.00	1.00
Median	0.00	0.00	-699.5	50.00	0.00	5.50	0.00	11.00
Mean	0.31	-2511	-15107.2	107.97	47.01	94.86	7.082	21.29
3rd Qu.	1.00	0.00	0.00	152.25	39.00	142.00	6.000	26.00
Max.	1.00	0.00	0.00	647.00	717.00	944.00	118.00	237.00

The target variable is default or non-default of the client defined as follows: default means the non-fulfillment of loan payment installments for 3 months in a row.

## 4. Results

In this section, we discuss the results obtained. The data set was divided into 2 portions: training and testing. Seventy percent of the data was used for training and the remaining 30% for the test. The data in both samples were distributed as follows: 37% defaulting and 63% non-defaulting. The target variable is the default status indicated with the value 0 for the non-default and with 1 the default.

We recall that the starting dataset presented 400 observations and 53 variables. The issue regarding the high number of variables with regards to the number of observations has been overcome by selecting the most significant variables through the Kruskal–Wallis test. The variables selected after applying the test are 21 and reported in the following list: previous funding, number revenue month 1, number releases month 1, number movements month 1, number revenue month 3, number releases month 3, number movements month 3, number revenue month 6, number releases month 6, number movements month 6, number revenue month 12, number releases month 12, number movements month 12, salary, total output essential goods, total financial services and utilities, frequency output non-essential goods, frequency output essential goods, frequency financial services and utilities, frequency salaries, frequency income. The model chosen for the credit scoring analysis is lasso logistics which represents an efficient choice in data analysis problems like ours when a variable selection step is needed.

For greater accuracy of the metrics obtained, we conducted the analysis using k-fold cross validation which partitions the dataset in subsets of equal size, where each subset is used as a test and the others as training. Moreover, we have conducted an out of sample analysis, training models on 2014-2015-2016 data and testing them on 2017 and 2018.

Parameters estimates of the 3 fitted logistic lasso models are reported in Tables 3–5 referred respectively to financial variables, textual variables and the mixed one. In particular, from Table 5 we can infer that several variables both financial and textual are significant. The textual ones, of course, are of major interest being the new ones. We observe that the largest parameter is obtained by the salary flag variable: having a negative sign means that the presence of the salary on the bank account decreases the probability of default. In particular if we calculate the odds ratio we get that the probability of non-defaulting is 12 times higher than the probability of defaulting. Thus, such a simple information, that can be derived by the analysis of bank transactions, can add very useful information to the credit holder. Other two textual variables are worth of mentioning: frequency of income characterized by a negative sign and the total output for financial services and utilities with a positive sign. According to the former the larger is the frequency in the income the lower is the probability of default. On the other way around, a higher number of transactions for financial services and utilities increases the probability of default. This to say that having several incomes helps in affording financial loans but the impact of expenses for services and utilities is not negligible. Regarding purely financial variables, all of them but one shows negative signs, meaning that they reduce the probability of default. The three largest parameters are shown by “previous funding” “number of movements at month 1” and “number of revenue at month 1.” What affects largely the chance of repaying loans is the presence of previous loans request to the bank. This ensures a previous capability of respecting financial obligations. The same applies to the number of movements in the nearest month: having more movements can be considered a symptom of financial health as if we take into account the number of revenues.

**Table 3:** Important variables selected by Lasso Model on financial dataset.

**Variables**	**Parameter estimated**
Previous funding	-0.4548
Number revenue month 1	-0.1553
Number releases month 1	·
Number movements month 1	-0.1759
Number revenue month 3	·
Number releases month 3	-0.0000
Number movements month 3	-0.0150
Number revenue month 6	·
Number releases month 6	-0.0000
Number movements month 6	-0.0001
Number revenue month 12	0.0028
Number releases month 12	·
Number movements month 12	·

**Table 4:** Important variables selected by Lasso Model on textual dataset.

**Variables**	**Parameter estimated**
Salary flag	-2.5998
Freq. salaries	·
Freq. income	-0.1111
Total output essential goods	0.0015
Total output financial services and utilities	0.0006
Freq. output non essential goods	-0.0083
Freq. output essential goods	·
Freq. output financial services and utilities	-0.1294

**Table 5:** Important variables selected by Lasso Model on mixed dataset.

**Variables**	**Parameter estimated**
Previous funding	-0.4984
Number revenue month 1	·
Number releases month 1	·
Number movements month 1	·-0.1650
Number revenue month 3	·
Number releases month 3	-0.0020
Number movements month 3	-0.0240
Number revenue month 6	·
Number releases month 6	·
Number movements month 6	·
Number revenue month 12	·
Number releases month 12	-0.0007
Number movements month 12	-0.0012
Salary flag	-2.5011
Freq. salaries	·
Freq. income	-0.0022
Total output essential goods	·
Total output financial services and utilities	0.0003
Freq. output non essential goods	·
Freq. output essential goods	·
Freq. output financial services and utilities	·

**Figure 1:**
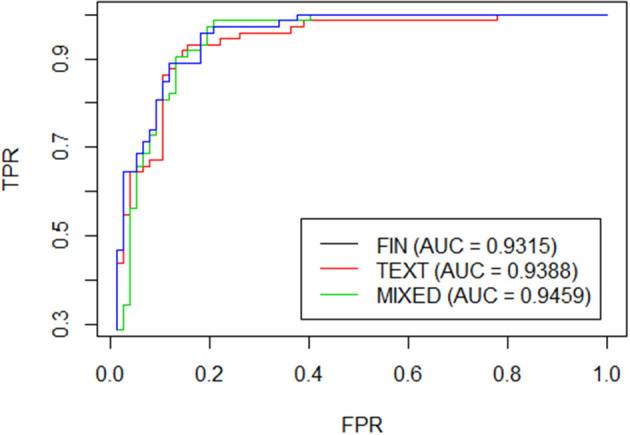
ROC curve.

Finally in Table 6 we report the comparison among the 3 models: the first which considers only the financial variables, the second which considers only the textual variables and the third one which combines both. The comparison is measured through Accuracy, Auc, type 1 error and specificity. As can be seen from Table 6, the results obtained are pretty high for the three models and even the roc curves tend to overlap many times as shown in Figure 1. If we focus on the accuracy, it is interesting to note that the model restricted to the textual variables component overcomes largely the model with only financial variables (84 vs. 75%). As a consequence the mixed model is an average of the two. On the other hand, the models are perfectly comparable in terms of AUC.

**Table 6:** Results from Lasso logistic regression.

	**Accuracy**	**Auc**	**False positive rate**	**Specificity**
Financial features	0.75	0.931	0.479	0.521
Text features	0.84	0.939	0.233	0.767
Mix	0.80	0.946	0.356	0.644

Nevertheless, there are important elements, first of all, classification errors. As you can see from Table 6, the mixed model and the text based one have an improvement over the type 1 errors. Not all errors have the same impact, some mistakes have higher implications than others. For a bank, type I error is the most dangerous one as it represents the probability of giving a loan to those who will not pay. The costs associated with type I errors are higher than type II errors.

## 5. Conclusions

In this paper, we use textual data to enhance the traditional credit scoring model. We evaluated the models performance by comparing the basic model in which only financial variables were included, against one in which there are only those extracted through text mining and the last one containing the mix of the two types of variables. From the analysis, we conclude that the addition of textual variables is relevant in the model. Although accuracy and AUC do not vary much, it should be emphasized first of all the distribution of errors. We observe an improvement over type 1 error and that both the textual based model and the mixed one show better results regarding accuracy. This is a promising result that encourages to further test the methodology with a larger dataset.

We can, therefore, state that despite the small size of the dataset, the analysis carried out shows how the textual analysis can be used in a credit scoring model to improve its accuracy.

Future research perspectives concern the application of the model to a larger dataset not only in terms of observations but also of variables based on textual information. More data can offer other types of information not available in the data at hand. Moreover the application of other text analysis technique like topic modeling (Cerchiello and Nicola, 2018) rather than the creation of manual dictionaries to make the process more automated and therefore decrease the time spent in pre-processing, can improve even more the quality of the analysis.

## Data Availability Statement

The data analyzed in this study was obtained from Moneymour and it is restricted by a non-disclosure agreement. Requests to access these datasets should be directed to RS, roberta.scaramozzino01@universitadipavia.it.

## Author Contributions

The paper is the product of full collaboration between the authors, however PC inspired the idea, the methodology and wrote sections Result and Conclusion. RS run the analysis and wrote sections Introduction, Methodology, and Data.

### Conflict of Interest

The authors declare that the research was conducted in the absence of any commercial or financial relationships that could be construed as a potential conflict of interest.
